# New Challenges in Sexuality and Sexual Dysfunction

**DOI:** 10.3390/jcm12010203

**Published:** 2022-12-27

**Authors:** Angel L. Montejo

**Affiliations:** University of Salamanca, Faculty of Nursing and Institute for Biomedical Research of Salamanca (IBSAL), Psychiatry Service, Clinical Hospital, Avenue of Donantes de Sangre SN, 37007 Salamanca, Spain; amontejo@usal.es; Tel.: +34-639754620

Human sexuality constitutes not only a basic need but also a right that significantly enriches interpersonal relationships, providing mutual satisfaction and pleasure. It undoubtedly contributes to quality of life and overall physical and mental health by improving self-esteem.

These dysfunctions and their consequences on a deteriorating emotional life and on the appearance of some mild to severe sexual difficulties and pathologies should be researched, understood and considered in treatment approaches as relevant aspects of general health.

Healthy human sexuality undoubtedly improves physical and mental health but during a pandemic, such as the COVID-19 (SARS-CoV-2) pandemic, major challenges need to be overcome or at least carefully analysed. Different situations involve different recommendations by experts in maintaining different types of sexual relationships without or minimizing health risks. To avoid contagions, such as during a pandemic, it is advisable to abstain from any type of sexual activity as a couple, replacing it with masturbatory activity or virtual sex instead, especially in patients with symptoms of COVID-19, in health professionals who are in contact with such patients or during pregnancy [1].

Special precautions must be taken to maintain an active sex life until the end of the quarantine period of a pandemic.

Many uncertainties regarding the differences between men and women in many aspects of sexual life remain to be understood. One of them is compulsive sexual behaviour disorder, which continues to be more frequent in men, although neuroticism and stress vulnerability seem to be more frequently reported in women [2]. More research is needed to understand in depth not only the differences but also the best approaches to overcoming these disorders when they constitute a barrier to the understanding and enjoyment of sex.

One group that is garnering growing research interest is the LGBTQ community, specifically, how lesbian, gay, bisexual, transgender, and queer people are affected by mastectomy for breast cancer [3]. Differential characteristics have been described in these different populations and must be considered according to patient preferences when making such decisions in surgical treatment processes.

One of the biggest challenges in sexuality research is the adolescent population, who need more attention, especially those who are victims of sexual violence. A high association has been found between adolescents who do not use condoms and with a background of any type of sexual violence; with behavioural problems; and who have higher levels of mental health illnesses, such as depression and anxiety, and lower levels of self-esteem [4]. Being older, male, and/or bisexual; reporting more frequent substance use; being non-Muslim; and reporting the use of the media to obtain sexual information were found to have the highest associations. Knowledge of this profile in adolescents could help to obtain better methods to prevent abuse. On the other hand, pornography use in adolescents can be seen as an anomalous way of understanding healthy human sexuality, and it would be necessary to insist on the need for adequate sexual training during school ages.

The elderly is one of the populations that receive the least attention in the study of sexuality. Most such studies have been performed in women, mainly during menopause, while in men, studies on erectile dysfunction predominate. The religious and social beliefs of this population certainly influence the encountered difficulties and greatly limit sexual behaviour in older adults. As sexuality has become widespread within the community in the twenty-first century, we will undoubtedly witness a redefinition of the importance of maintaining a healthy and prolonged sex life, especially in women who will abandon the traditional role of providing men with sexual pleasure to seek an experience of shared and rewarding pleasure.

Sexual desire is one of the most frequent dysfunctions related to sexual health, especially in women, although its differences in men have not been investigated much. Among women, interpersonal issues and low physical attraction were highly related to their sexual interest, and physical attraction and daily hassles in males were high predictors of low sexual desire in females. Women looked for sex therapists and psychological interventions more often than men, and both viewed gynaecologists as a more acceptable therapist than a general practitioner [5].

Research on sexuality cannot neglect to study the possible iatrogenesis caused by some frequently used drugs such as antidepressants [6], antipsychotics [7], and antihypertensives [8]. The data have been corroborated not only in patients but also in healthy volunteers [9]. These drugs affect desire, orgasm, and sexual arousal, effects that go unfortunately unnoticed if the appearance of their symptoms is not systematically explored. Through the PRsexDQ-Salsex (Psychotropic-Related Sexual Dysfunction Questionaire—Salsex) [10], a validated questionnaire for the measurement of sexual dysfunction by psychotropic drugs and, by extension, all medications, it has been found that very low spontaneous communication (6.81% females/24.8% males) is seen in patients, although 66.40% of the patients reported moderate to severe impaired sexual function after the use of antihypertensive compounds [8]. A less deteriorating compound was angiotensin II receptor antagonists (ARBs) (29.8%), in comparison with combined treatments, mainly diuretic + ARB (74.2%). These differences should certainly play a primary role in choosing an antihypertensive drug for use in a sexually active person. On the other hand, effective management strategies are needed to face this problem and to serve as a practical guide for clinicians who frequently prescribe these psychotropic drugs to be of great help in patients who need them but to avoid unnecessary prescriptions [11].

On the other hand, sexual function is impacted by chronic diseases, with different impacts by each, and this has been studied sparsely. An example is the impairment of sexual function in women experiencing symptoms of endometriosis, including sexual pain and dyspareunia. Another example is an anal fissure, which is usually accompanied by poorer quality of life (QoL) and sexual dysfunction together with anxiety and depression. Recent data on the impact of hormone therapy in females with primary adrenal insufficiency (PAI) have been obtained and showed worse scores in desire, arousal, lubrication, and overall sexual satisfaction compared with the controls [12]. Additionally, isolated hypogonadotropic hypogonadism is related to decreases in patient mood, sexual satisfaction, and health-related quality of life. The relationship between sexual problems experienced by treatment and a high non-compliance rate (51.6%) stands out. Another example is the prevalence of autoimmune thyroid disease (AITD), where in females, hypothyroidism influences libido and sexual excitability, with, nevertheless, a low influence on orgasms. Sexual dysfunction worsened with higher levels of depression and higher TSH values [13]. As a new approach, the exploration of sexual function present in organic pathologies, often underestimated, should be performed routinely to improve treatment goals, adherence, and quality of life.

Research on sexual function benefits from the emergence and validation of new and better questionnaires that complement existing ones, such as the Center of Applied Psychology Female Sexual Questionnaire (CAPFS-Q) [14], including the following aspects: sexual relations with a partner, sexual practices, and dysfunctional aspects of sexual relations. The DESEA Questionnaire [15] was recently created and adequately validated to identify hypoactive sexual desire disorder. All new questionnaires need to be short and easy to administer.

Although it is already known that mental health, specifically anxiety levels, has a decisive influence on erectile dysfunction (ED), it has been found that high levels of anxiety significantly delay erectile response to alprostadil intracavernous injection (ICI) [16]. This late response to alprostadil could help in the diagnosis of non-organic ED using a penile echo-colour Doppler ultrasound (PCDU).

A tremendously shocking fact that has hardly been studied is that genital mutilation/cutting (FGM/C) leads to great physical and psychological dysfunctions. After reconstructive surgery in 15 women, sexual distress, general psychopathology, and genital self-image were significantly improved, but unfortunately, sexual function was not improved [17].

Sexuality is constantly evolving as society changes, and therefore, its expression in new and dangerous forms such as Chemsex, which involves participation in sexual relations under the influence of drugs, has increased in frequency in recent years. This increasing fact could lead to a serious public health issue, especially between persons diagnosed with the human immunodeficiency virus (HIV). A recent study analysed Chemsex in a group of 101 males with HIV who have sex with men, showing that 40.6% of them had practiced it during the last year. This group additionally presented more risky sexual behaviours, such as occasional and/or multiple sexual partners [18]. Finally, there was a negative relationship between Chemsex and health-related quality of life, indicating the need to alert this population to the increasing health risks of this practice.

Undoubtedly, we have many new challenges to overcome in terms of sexuality being understood as a health good that must be addressed and researched by health professionals to ensure adequate quality of life. However, one of the biggest concerns worldwide for reaching adequate sexual health is the lack of properly trained professionals who can contribute to the detection of dysfunctions to thus improve the sexual health of populations while mainly working in public health systems. If sexuality remains considered a luxury and not as a relevant aspect of global health that must be cared for and preserved and if health systems do not adequately consider it as such, a unique opportunity to treat medical and psychic pathologies as an inseparable whole, constituting a true wholistic approach to health, will be lost.
